# Extraskeletal Chondroma of Anterior Abdominal Wall in a Child

**Published:** 2013-12-01

**Authors:** Prashant Goyal, Shelly Sehgal, Soumyesh Ghosh, Deepti Mittal, Sompal Singh

**Affiliations:** Department of Pathology, Swami Dayanand Hospital, Dilshad Garden, Delhi, India

A 7-year-old boy presented with a slightly raised, painless swelling on left lower quadrant of anterior abdominal wall. The swelling was slowly growing for 10 months. There was no history of preceding trauma and no relevant past medical and surgical history. General physical and systemic examination did not reveal any significant findings. On local examination, there was a non-tender, firm to hard nodular swelling of 2.0 cm x 1.5 cm in size over the anterior abdominal wall, free from overlying skin and underlying muscles. There were no other subcutaneous masses. His routine laboratory investigations were within reference range. The mass was excised and sent for histopathological examination. Grossly, the cut surface was firm, blue-white and glistening. Hematoxylin and eosin stained sections showed confluent lobules of hyaline cartilage with variable cellularity comprising of mature chondrocytes embedded in chondrocytic lacunae (Fig. 1, 2). There was no significant cytologic atypia, mitosis, or necrosis. No calcification/ ossification were present throughout the mass. Final diagnosis of extraskeletal chondroma (ESC) was made.

**Figure:**
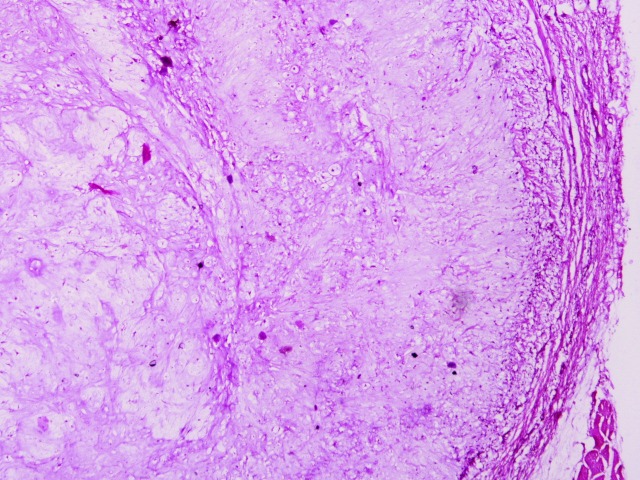
Figure 1: Well-circumscribed tumor showing lobulated appearance of hyaline cartilage (x40).

**Figure:**
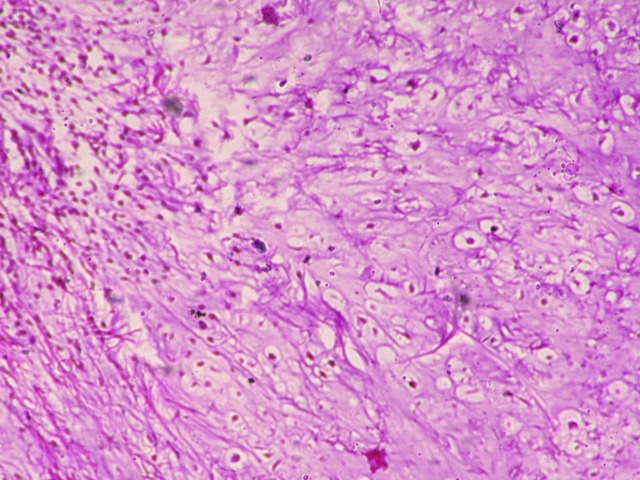
Figure 2: Hyaline cartilage consisting of bland appearing chondrocytes in lacunae (x100).

## DISCUSSION

ESCs are benign mesenchymal soft-tissue tumor composed mainly of hyaline cartilage without any bony or periosteal involvement. It constitutes only 1.5% of benign soft-tissue tumors.[1,2] Most of ESCs were found between 30-60 year of age and have an equal sex distribution. It is very rarely seen in children.[2] ESC arise principally in extremities (96%) with 72% in the hands and 24% in the feet and only 4% in the rest of the body.[1,2] In our case, it was located over the anterior abdomen wall.

Clinically, they present as a slow-growing mass, occasionally causing pain or tenderness. They are usually less than 3 cm in size and firm or rubbery on palpation.[1] Soft-tissue masses of anterior abdominal wall in children encompass a wide variety of lesions including soft-tissue abscess, lipoma, posttraumatic hematoma, vascular malformation, desmoid tumor, and cysticercosis. The etiology of ESC is uncertain. It is believed that either these lesions develop from residual embryonal tissue in the area of preexisting fetal cartilage or pleuripotent mesenchymal cells undergo metaplasia, differentiating into cartilage.[2] Malignant transformation has not been reported in English literature.[3]

On plain radiographs, ESC may appear as well-circumscribed, lobulated mass with dense central mineralization.[4] Calcification is usually ring-like punctate or granular. Sometimes, mineralization has an unusual configuration or is completely absent. The lesion should be differentiated from myositis ossificans, tumoral calcinosis, chondroid syringoma, low-grade extra-osseous chondrosarcoma and extraskeletal osteosarcoma. Wide local excision is the optimal treatment for ESC. Local recurrence has been seen in up to 18% cases and metastasis has not been documented.[5]

## Footnotes

**Source of Support:** Nil

**Conflict of Interest:** None declared

